# A Method for Prediction of Ultrasonic Detectability of Interface Gap Defects on TC4 Diffusion-Bonded Joints

**DOI:** 10.3390/nano12060911

**Published:** 2022-03-10

**Authors:** Lichen Teng, Zhenggan Zhou

**Affiliations:** School of Mechanical Engineering and Automation, Beihang University, Beijing 100083, China; zzhenggan@buaa.edu.cn

**Keywords:** ultrasonic inspection, titanium alloy, diffusion bonding, acoustic response model, detectability

## Abstract

An analysis method for the detectability of defects on the TC4 (Ti-6Al-4V) diffusion bonding interface was proposed in this study. First, a semi-analytical model of the liquid–solid coupling acoustic field with attenuation characteristics was constructed. Based on this, a method for the selection of transducer parameters was investigated for effective focus on the diffusion bonding interface. Second, according to the characteristics of defects on the diffusion bonding interface, an acoustic response model for diffusion bonding defects was established based on Kirchhoff approximation. The detectability of defects on the diffusion bonding interface was analyzed using transducers of different frequencies with different diffusion bonding interface gaps. Finally, an experiment was conducted to verify the reliability of the simulation. The analysis method proposed shows the advantages in the selection of suitable parameters for detecting specific diffusion bonding interface gaps, providing theoretical predictions of the detectability of diffusion bonding interface defects.

## 1. Introduction

Diffusion bonding is a solid-phase welding process, by which two prepared surfaces are joined at elevated temperatures under applied pressure [1]. Due to the complexity of the diffusion bonding process, factors such as surface roughness of the workpiece material and contamination or improper selection of the diffusion joining process parameters will cause defects in the diffusion bonding interface. These defects, such as unbonded and interfacial microvoids, seriously reduce the mechanical properties of TC4 alloy diffusion bonding components [2], such as those of the hollow blades of aeroengines [3,4,5]. Considering the requirements for high reliability and safety in the aerospace field, reliable nondestructive testing for TC4 diffusion bonding joints is required.

Currently, the ultrasonic testing method is more suitable for diffusion-bonded joints, since it has higher sensitivity in comparison to X-ray methods [6]. Among the ultrasonic technologies, the ultrasonic C-scan inspection technology is the most widely used in the nondestructive evaluation of diffusion bonding [7]. By using this technology, the defects and their distribution on the diffusion bonding interface can be easily obtained; then, the strength of the diffusion bonding interface can be connected with ultrasonic wave reflection of the defects. Previous studies have been conducted on the applications of ultrasonic C-scan inspection in diffusion bonding. Kumar et al. [8] used water immersion ultrasonic inspection to detect cylindrical Ti-6Al-4V diffusion bonding specimens. The ratio between diffusion bonding interface echo and surface echo was measured, and it was used to establish the correlation with the defects on the diffusion bonding interface of different areas. Lavrentyeva et al. [9] used the stiffness coefficient of the diffusion bonding interface to establish the quantitative correlation between the ultrasonic reflection coefficient and the microscopic size of the interface. Liu et al. [10] used a high-frequency ultrasonic transducer to detect titanium alloy thin plates and believed that increasing the test frequency is an effective method for detecting diffusion bonding interface defects. Edwill et al. [11] used the nonlinear effect of ultrasonic around the diffusion bonding defect and detected a diffusion welding defect with a thickness of 12.7 mm on one side of the sample. Luan et al. [12] used transducers with different frequencies to conduct inspection experiments on TC4 alloy diffusion bonding circular test blocks. They concluded that the detection of defects on the diffusion bonding interface requires a reasonable selection of transducer parameters and the full consideration of relevant signal processing methods. However, they did not obtain a method to select the parameters of the transducer.

As observed from recent studies, ultrasonic inspection technology has been widely applied to test defects on diffusion bonding interfaces. Most research work did not have a standard for the selection of process parameters. For weak diffusion bonding interface gaps, to reduce the risk of false detection of such defects, it is important to carefully select the parameters of ultrasonic inspection processes.

In this paper, an acoustic model was established to describe the weak gap in diffusion bonding interface defects. Based on the wave equation, the Rayleigh–Sommerfeld integral general formula was used to obtain a semi-analytical displacement field expression of the focusing transducer in the liquid–solid coupling media. A simulation model of the internal acoustic field of a TC4 alloy with the attenuation coefficient was constructed. Then, a selection method for the parameters of transducers was developed by modeling the acoustic field. The acoustic attenuation coefficient of the thick TC4 alloy diffusion bonding base material was measured Then, based on the acoustic field simulation model, the selection of detection parameters of the transducer completed. Furthermore, a diffusion bonding interface defect-response model was established. Based on this model, the correlation curve between the diffusion bonding interface gap of diffusion welding and the detection frequency of the transducer was established to predict the detectability of diffusion bonding interface defects with different diffusion bonding interface gaps. The results of corresponding experiments show that the proposed method can provide a theoretical basis for the analysis of the detectability of diffusion bonding interface defects.

## 2. Experimental Setup

### 2.1. Manufacture of Diffusion-Bonded Samples

The diffusion-bonded sample is made by joining two 120×40×10 mm TC4 blanks of 120×40×10 mm. The 120×40 mm faces of the blanks were grounded to a finish of 0.2 μm Ra. Air was evacuated from the interface and the samples underwent a hot isostatic process.

**Figure 1:**
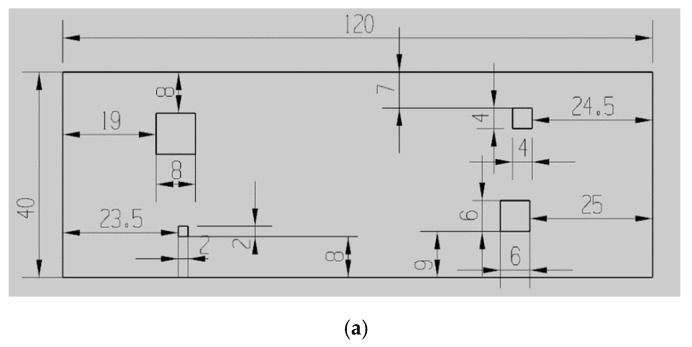

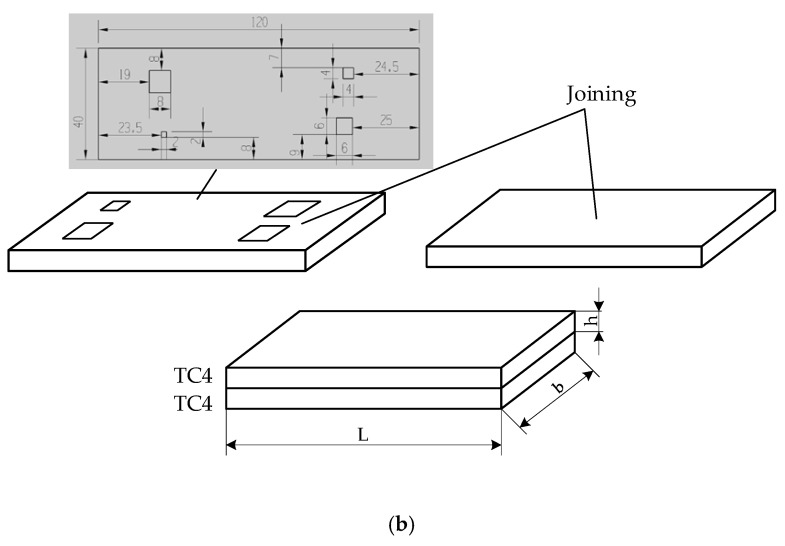
Schematics of sample defects. (**a**) Arrangements of the sample defects, (**b**) Production process of sample defects.

To obtain different diffusion bonding interface gaps, ultrafast laser femtosecond technology was used to make square grooves with a depth of 20 μm on one TC4 blank, and their side lengths were 2 mm, 4 mm, 6 mm, and 8 mm, respectively (See Figure 1a). Then, the blank was joined with the other one, for which its faces were not grooved (See Figure 1b). The production process of the sample defects is shown in Figure 1.

### 2.2. Ultrasonic Inspection and Cut-Up

In order to verify the results of the numerical calculation, an experimental setup with the configuration of inspection parameters for the generation and detection of ultrasonic waves was established to investigate the reflected waves and diffusion bonding joints.

An ultrasonic emission acquisition module was utilized to induce ultrasonic waves. An acquisition board was used to collect the data of the received signal. The position of the transducers could be adjusted by the six-degree-of-freedom robot. The trajectory of the diffusion sample was accurately controlled by a program, and their detailed parameters are shown in Table 1.

**Figure 2:**
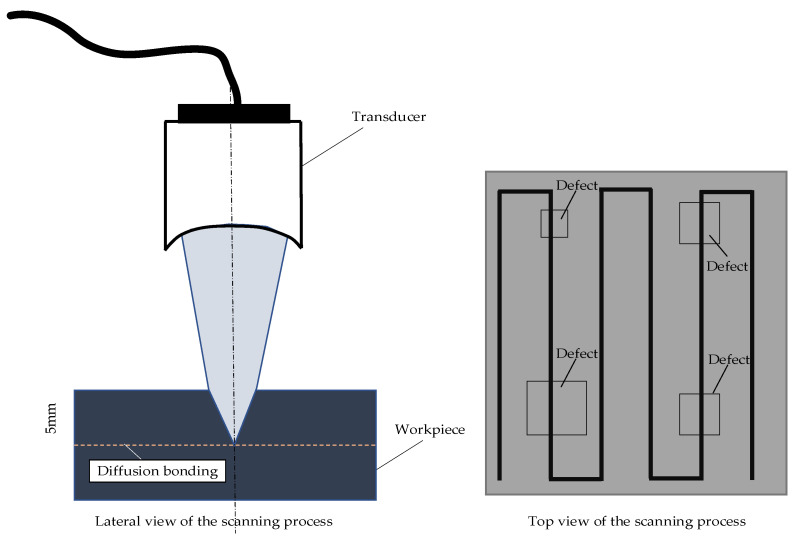
The ultrasonic testing position schematic diagram and top view of the canning process of the sample.

A diffusion-bonded sample with an area of 120×40 mm was ultrasonically scanned in pulse echo, with the probe focused at the diffusion bond depth (5 mm). The test was conducted with a scanning step of 0.1 mm to obtain a high-resolution inspection result. The parameters of the transducer can be observed in Table 1, and the ultrasonic testing position of the sample is shown in Figure 2.

## 3. Results and Discussion

### 3.1. Analysis of Characteristics of the Acoustic Field Radiated by Focusing Transducer

By an ultrasonic flaw detector, the second and third echo amplitudes at a specific position of the specimen (30 mm) were collected. Based on Equation (1), attenuation values were calculated.

The acoustic velocity of the TC4 alloy, $c_{2}$, and the ultrasonic attenuation coefficient, β [13], can be described by the following equation:

$$c_{2}=\frac{t_{B_{2}}-t_{B_{1}}}{2H}c_{1}$$

(1) $$\beta =\frac{10\mathrm{lg}\left(\frac{B_{F1}}{B_{F2}}\right)}{H}$$

where $B_{F_{1}}$ and $B_{F_{2}}$ represent the amplitude value of the second and third bottom echoes; H represents the thickness of the specimen; $t_{B1}$ represents the received time of the first bottom reflection wave; and $t_{B2}$ represents the received time of the second bottom reflection wave.

The attenuation coefficient is shown in Table 2.

**Figure 3:**
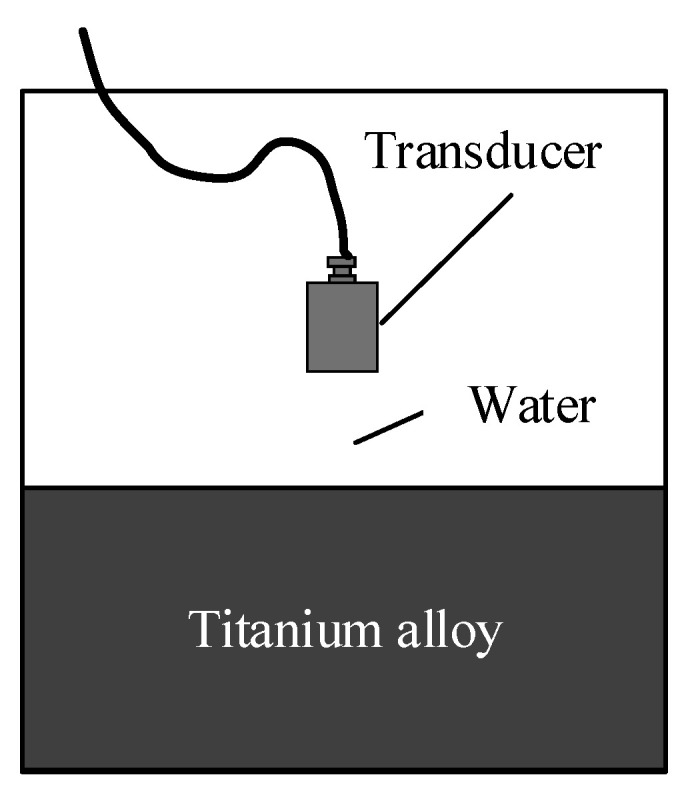
The acoustic field model.

Based on the theoretical model of the acoustic field of the focusing transducer under liquid–solid coupling (as observed from Figure 3) according to the attenuation value, the water acoustic speed, the TC4 acoustic speed, and the density, the grid discrete accuracy is set to be λ/10 and is calculated by the following:

$$\lambda =\frac{5.063\times 10^{3}}{f}(\mathrm{mm})$$

where f was set to be 5 MHz, 10 MHz, and 15 MHz, respectively. The grid type was set to be quadrilateral. Thus, the amplitude of different parameters of the transducer, including the diameter and focal length, can be calculated for the acoustic field simulation.

To study the distribution of the acoustic field in the TC4 alloy with different process parameters, the influences of various factors on the acoustic field of the transducer were analyzed by adjusting the frequency, diameter, focal length, and other parameters of the transducer.

**Figure 4:**
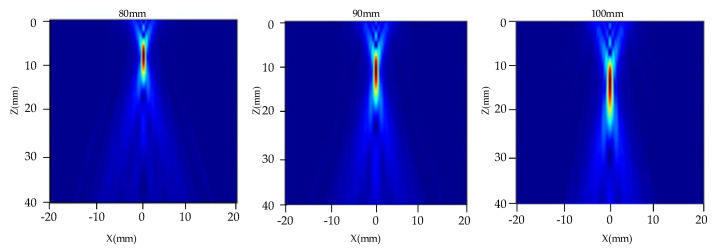
Distribution of acoustic field radiated by transducers with different focal lengths.

Figure 4 shows the two-dimensional acoustic field distribution in the TC4 alloy diffusion bonding plate. To study the influence of the transducer focal length on the radiated acoustic field, a transducer with a frequency of 10 MHz and a diameter of 19 mm was used. The focal lengths were set to be 80 mm, 90 mm, and 100 mm, and their acoustic field distribution can be observed in Figure 4, respectively. When the focal length of the transducer increases, the acoustic beam moves downward as a whole. Although the length of the near-field in the sample increases, it is beneficial for detecting the defects in the deeper position.

**Figure 5:**
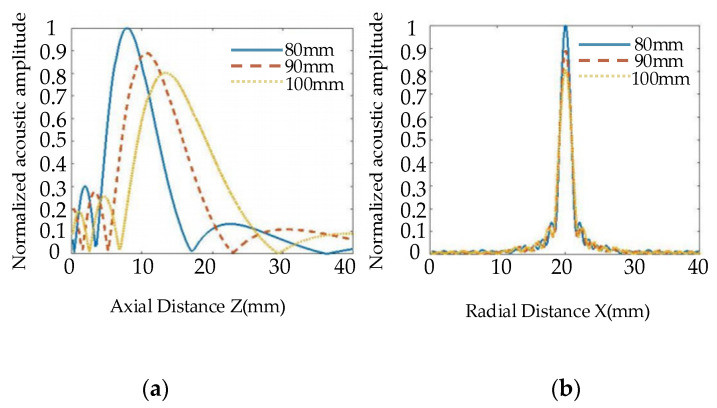
One-dimensional axial acoustic pressure distribution of transducers with different focal lengths. (**a**) Axial acoustic pressure, (**b**) Radial acoustic pressure at a focal plane.

Figure 5 is a one-dimensional axial acoustic pressure distribution curve. It can be observed from Figure 5a that, as the focal length of the transducer increases, acoustic pressure decreases. The slower the acoustic pressure decreases at the same axial distance, the larger the focal column size will be. According to the radial acoustic pressure curve of the focal plane (See Figure 5b), the focal length of the transducer hardly affects the focus of the transducer, and the focal spot size remains unchanged.

**Figure 6:**
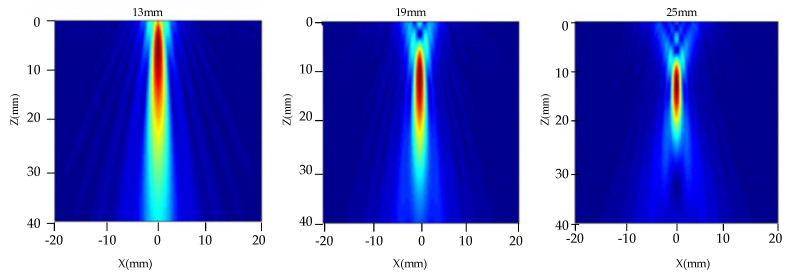
Distribution of acoustic field radiated by transducers with different diameters.

Figure 6 shows the effect of the transducer diameters on the radiated acoustic field. A transducer with a frequency of 5 MHz and a focal length of 100 mm was selected. The diameters were set to be 13 mm, 19 mm, and 25 mm, and their acoustic field distribution can be observed in Figure 6, respectively. The distribution of the radiated acoustic field in the TC4 alloy with a water distance of 50 mm was analyzed. It can be observed from Figure 6 that, as the diameter of the transducer increases, the length of the main lobe of the acoustic beam decreases and the side lobes decrease. The radiated acoustic beam becomes narrower, and the energy is more concentrated. The acoustic beam moves downward as a whole, the near-field length of the focus transducer increases, and the focal length increases, which is beneficial for detecting deeper defects.

**Figure 7:**
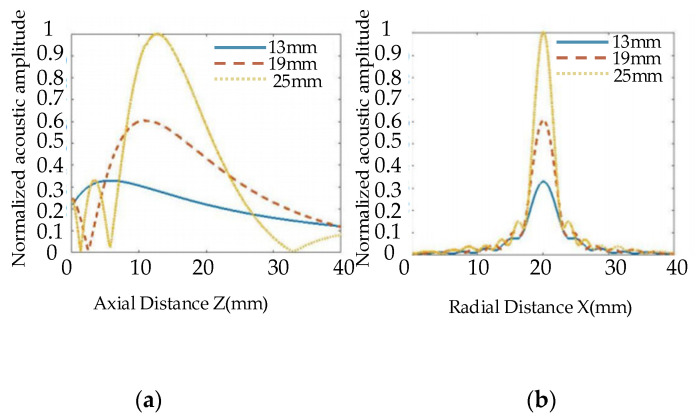
One-dimensional acoustic pressure distribution of transducers with different diameters. (**a**) Axial acoustic pressure, (**b**) Radial acoustic pressure at a focal plane.

Figure 7 shows the acoustic field axis and the distribution curves of radial acoustic pressure on the focal plane of three transducers with different diameters. The ordinate is the normalized relative amplitude of the acoustic field of the three transducers. It can be observed from Figure 7a that, as the diameter of the focusing transducer increases, the near-field length of the acoustic field becomes longer. As the focal length increases, the maximum acoustic pressure increases. As the acoustic pressure drops more at the same distance, the acoustic pressure attenuation is faster. It also can be observed from Figure 7b that, as the focal column becomes smaller, the focal spot becomes smaller. The acoustic energy on the axis is increasingly concentrated relative to the focal point of the acoustic beam. This demonstrates that the large probe has better directivity and higher resolution than the small probe. Small defects are easier to detect at higher resolutions, but the near-field length increases at the same time.

**Figure 8:**
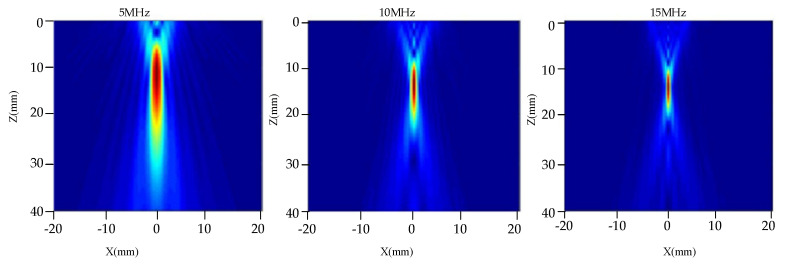
Distribution of acoustic field radiated by transducers with different frequencies.

Figure 8 shows the influence of transducer frequencies on the distribution of the radiated acoustic field. Transducers with a fixed diameter of 19 mm and a focal length of 100 mm were selected. The frequencies are selected to be 5 MHz, 10 MHz, and 15 MHz. Their acoustic field distribution can be observed in Figure 8, respectively. The distribution characteristics of the acoustic field with a water distance of 50 mm were analyzed. It can be observed from Figure 8 that as the frequency of the transducer increases, the number of main lobes decreases, and the acoustic pressure spreads less. The amplitude distribution of the main lobe in the far field decreases as the frequency increases. As the frequency of the transducer increases, the near-field length and focal length become longer, and the depth that the focusing transducer can detect becomes greater. This shows a similar trend to the influence of the diameter of the probe.

**Figure 9:**
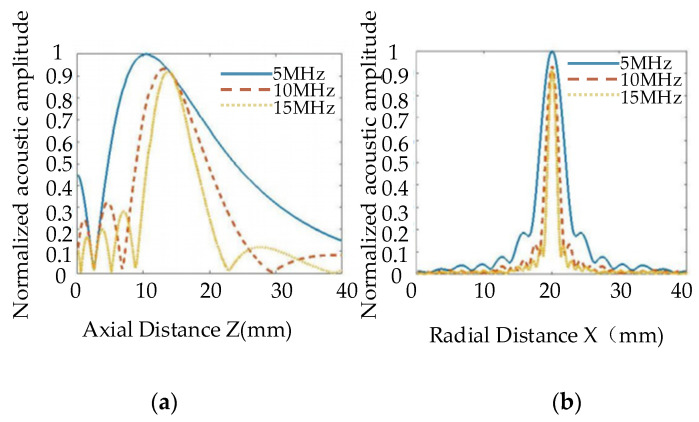
One-dimensional acoustic pressure distribution diagram of different frequency transducers. (**a**) Axial acoustic pressure, (**b**) Radial acoustic pressure at a focal plane.

Figure 9 shows the distribution curves of one-dimensional axial acoustic pressure (a) and radial acoustic pressure on a focal plane, (b). We can observe from Figure 9a that, as the emission frequency of the probe increases, the near field becomes longer, and the focal column becomes smaller. At the same time, we can observe from Figure 9b that, as the focal spot becomes smaller, the focal length increases, the acoustic pressure decreases slightly, and energy becomes more concentrated. Therefore, higher frequencies would be more conducive to the detection of small defects.

### 3.2. Transducer Parameter Selection Method

Based on the conclusion of the transducer acoustic field simulation, we can observe that the transducer’s diameter and frequency mainly affect focal spot size: When the transducer diameter or frequency increases, the focal spot size decreases. The focal length of the transducer mainly affects the focal position. The longer the focal length, the deeper the focal position in the workpiece. Therefore, in actual nondestructive testing, a comprehensive consideration of various factors is required to obtain suitable inspection parameters.

We have concluded that the focal length of the transducer mainly affects the focal position. To ensure that the acoustic wave focuses on the diffusion bonding interface, the transducer focal length needs to be selected according to the sample’s thickness. Additionally, to ensure that the lateral detection resolution is sufficient, the focal spot size should be simulated by adjusting the transducer’s diameter and frequency. Therefore, for a sample with 10 mm height, to ensure that the acoustic wave was able to focus at 5 mm, we simulated a common transducer diameter of 13 mm. As we know from Figure 8 and Figure 9, a higher frequency is beneficial for a higher inspection resolution—a smaller focal spot. Here, we chose a frequency of 5 MHz for the simulation, although higher frequencies of 10 MHz, 15 MHz, and even 20 MHz can also meet the resolution.

**Figure 10:**
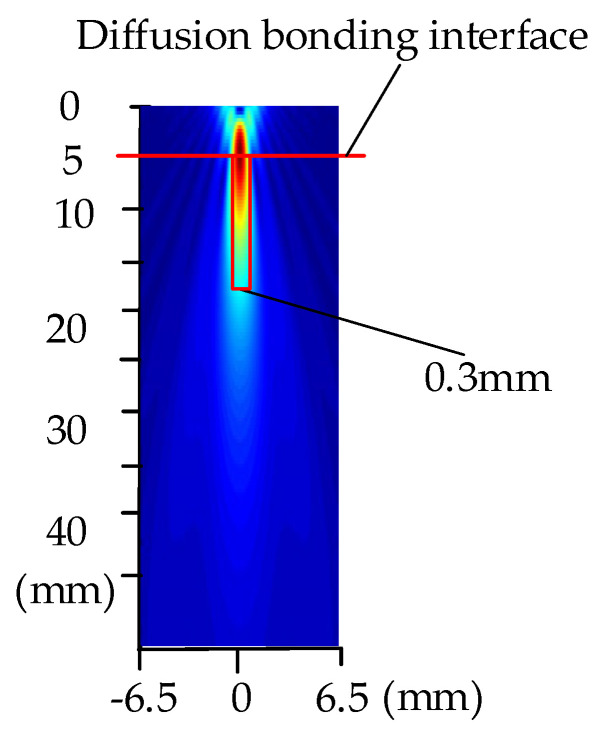
Distribution of acoustic field radiated by transducers with diameter of 13 mm, frequency of 5 MHz, and focal length of 50 mm.

Figure 10 shows the distribution of the acoustic field radiated by transducers with a diameter of 13 mm, frequency of 5 MHz, and focal length of 50 mm. We can observe from the figure that the highest energy of the main lobe in the acoustic field passes through the location of the diffusion bonding interface, which means that a focal length of 50 mm is able to focus at 5 mm of the sample. The spot size was approximately 0.3 mm, which required that the area of the defects be at least greater than 0.3 mm to ensure the inspection’s resolution.

### 3.3. Analysis of Acoustic Response of Diffusion Bonding Interface Defect

**Figure 11:**
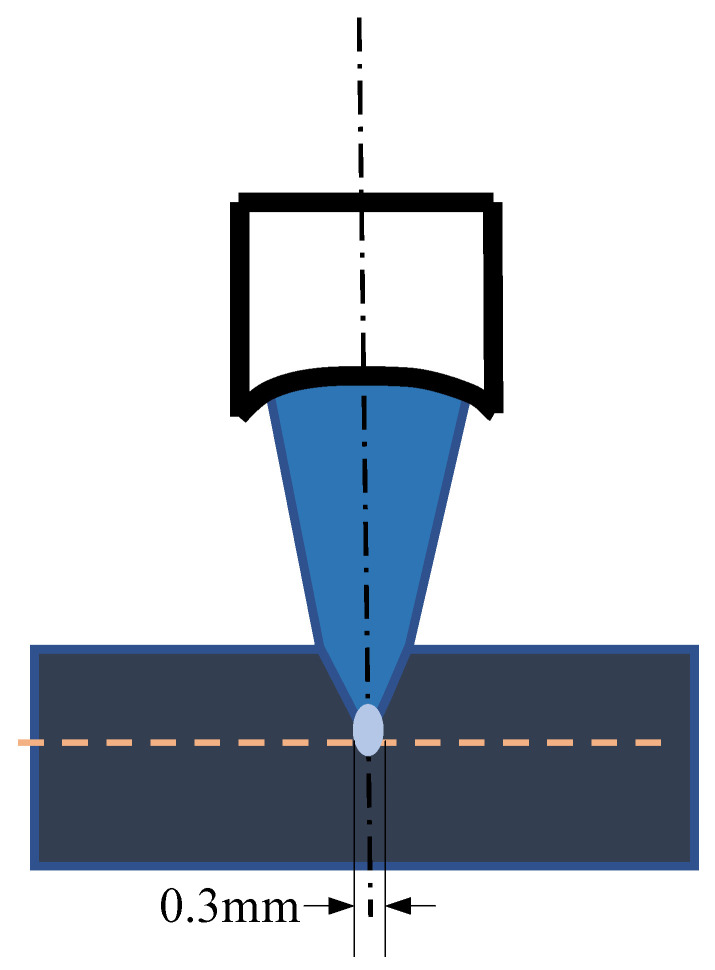
Schematics of the focal spot.

To discuss the correlation between different frequencies with diffusion bonding gaps, the main simulation frequencies of transducers were chosen to be 5 MHz, 10 MHz, 15 MHz, and 20 MHz. As observed in Figure 10, the transducer’s diameter was set to 13 mm. Using this parameter, the minimum focal spot size of the transducer was approximately 0.3 mm, as observed from Figure 11; thus, the lateral area of diffusion bonding interface defect should be larger than 0.3 mm.

**Figure 12:**
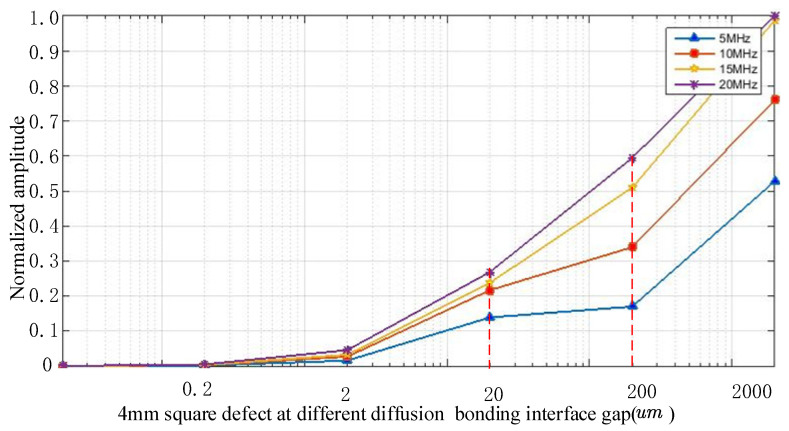
Curve of correlation between echo amplitude of the 4 mm square defect, the detection frequency, and the diffusion bonding interface gap.

Since the defects of diffusion bonding are mainly microvoids, their height (perpendicular to the bonding interface) comprises micrometer levels, and the cross-section is almost elliptical [14]. The defect scattering model based on Kirchhoff’s approximation has certain advantages in characterizing the scattered acoustic field of ellipsoidal defects. Therefore, the ellipsoidal defects in Kirchhoff’s approximate defect scattering model were used to simulate diffusion bonding interface defects. The ellipsoid defect model was first discretized, and then the specular reflection echo generated by each separate defect point was calculated according to Kirchhoff’s approximation. Then, the discrete value of the defect echo generated by all discrete points was superimposed to obtain the discrete value of the scattered echo of the entire defect. Based on this model, the scattering amplitude of longitudinal waves and ellipsoidal defects can be calculated. The specific calculation process is as follows:

(1) The surface of the focusing transducer was discretized into tiny cells, and the direction vector of the incident acoustic wave from each discrete point to the diffusion bonding interface defect was calculated. Then, Kirchhoff approximation scattering theory was used to obtain the scattering amplitude of each incident direction, and the echo scatter amplitude of the defect can be obtained.
(2) The diffusion bonding interface gap increased from 0.02 μm to 200 μm in 10-fold increments. The other two transverse semiaxes of the defect were, respectively, set to be 4 mm. According to the transducer detection parameters obtained by the simulation, the correlation curve between a 4 mm square defect at different diffusion bonding gaps and different frequencies was calculated, as shown in Figure 12. The selected transducer detection parameters are shown in Table 3.

As observed from Figure 12, there is no echo amplitude at the gap of 0 μm, and with an increase in the gap, the echo amplitude also increases, which meets the actual situation. If a diffusion interface is bonded well (The gap is 0 μm), there will be no echo amplitude. Regardless, as the diffusion bonding interface gap of the TC4 alloy diffusion defect becomes larger, the reflected echo of the diffusion welding defect continues to increase. At the same diffusion bonding interface gap, the higher the frequency, the stronger the reflected echo, especially at the diffusion bonding interface gap of 20 μm; the echo amplitude corresponding to a 15 MHz transducer can reach an amplitude close to 25%, which is nearly the same as that of a 20 MHz transducer. Therefore, it is theoretically feasible to use a 15 MHz transducer to detect diffusion bonding interface defects with a diffusion bonding interface gap of 20 μm.

### 3.4. Experimental Results

To verify the diffusion bonding interface defect response model, an experiment was conducted. During the inspection process, the peak imaging of the interface defect echo was used, and the C-scan image of the sample was obtained.

**Figure 13:**
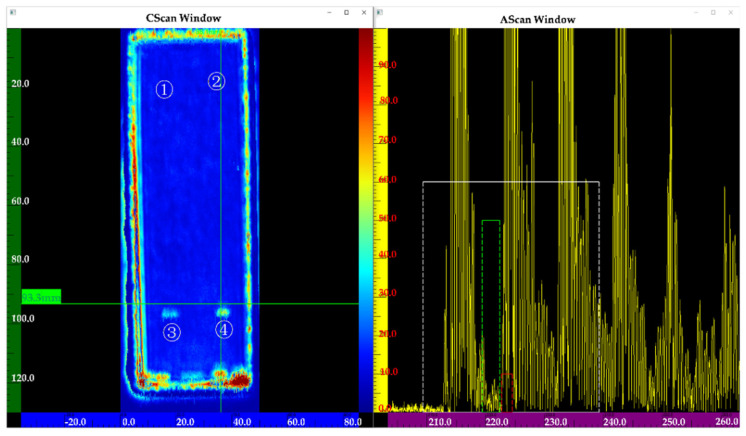
Inspection results using a frequency of 5 MHz.

**Figure 14:**
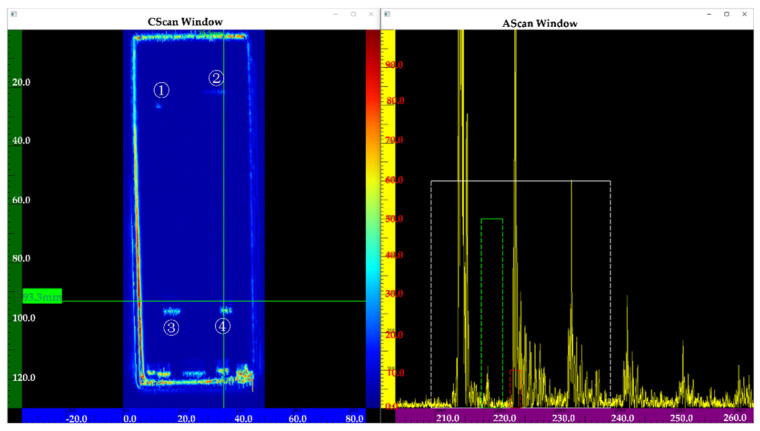
Inspection results using a frequency of 10 MHz.

**Figure 15:**
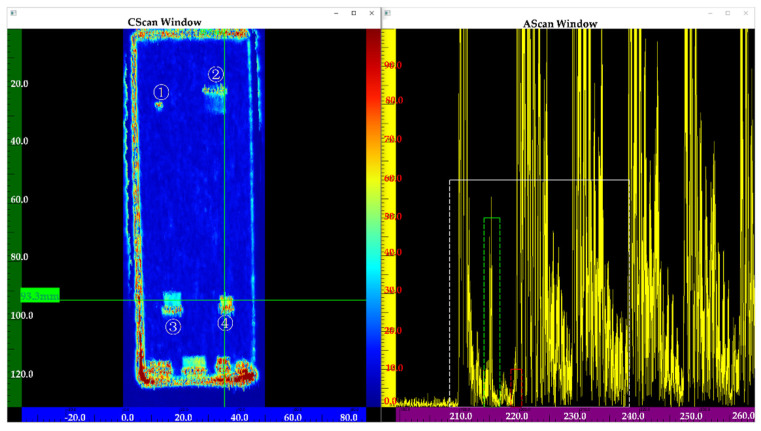
Inspection results using a frequency of 15 MHz.

Figure 13 shows the inspection results of the sample using a transducer frequency of 5 MHz. Flaws ③④ can be clearly observed in the C-scan image, but flaws ①② cannot be observed. Compared to Figure 13, Figure 14 shows the four flaws more clearly. In the A-scan images, the amplitude of a flaw at the same position (See Figure 14) is higher than in Figure 13. Although flaws ①② can be partly observed, they are not very clear (See Figure 13). Figure 15 shows a better result. In the C-scan image, all flaws can be observed, and the flaw shape is consistent with the predesigned defect shape. In a comparison of Figure 13, Figure 14 and Figure 15, it is obvious that for the defects with the same diffusion bonding interface gap, the higher the frequency, the greater the amplitude of the defect echo, which is consistent with the theoretical curve law (See Figure 12). This implies the reliability of the response model for the diffusion bonding interface defect.

**Figure 16:**
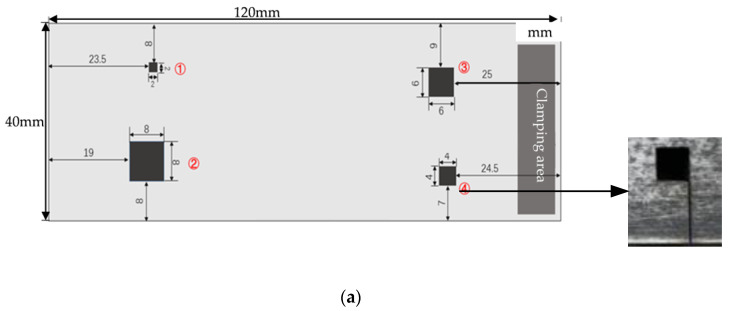

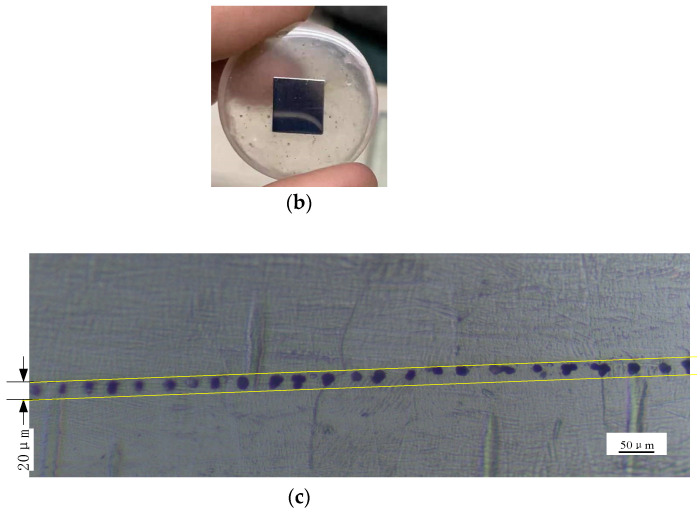
Metallurgical microscope picture. (**a**) Sample cut image ④, (**b**) Morphology of the diffusion bonding interface defect ④, (**c**) Morphology of the diffusion bonding interface defect ④.

To verify the effectiveness of ultrasonic testing results, defect ④ was processed and cut, and then the processed sample was polished and corroded. A 500-fold metallurgical microscope was used to conduct observation experiments on the dissected samples. The experimental results are shown in Figure 16. We can observe from the figure that the diffusion defect presents a series of elliptical interfaces under the metallographic microscope, which is consistent with the theoretical simulation results of the diffusion bonding interface defect shape. The size of the diffusion bonding interface gap was about 3~20 μm. This shows the sensitivity of ultrasonic detection for diffusion welding defects with the disengagement height.

## 4. Conclusions

This work proposed a method for predicting the detectability of diffusion bonding interface defects. An acoustic field simulation model under liquid–solid coupling with attenuation was established.

The influences of transducer diameter, focal length, and frequency of detection were theoretically analyzed. Then, a response model for the diffusion bonding interface defect was established with the Kirchhoff approximation. The correlation between the diffusion bonding interface gap and the transducer frequency was obtained. Corresponding experiments were conducted to verify the reliability of the transducer parameter selection method and the diffusion bonding interface defect response model. Finally, the following conclusions have been drawn:

(1) A transducer parameter selection method was established by the simulation model. The simulation results suggest that diameter and frequency both affect focal spot sizes and focal positions. When the transducer diameter or frequency increases, focal size decreases, and the focal spot moves upward. The focal length of the transducer mainly affects the focal position, and the size of the focal spot has a small effect. According to the simulation results, we can choose suitable transducer parameters for the diffusion bonding sample.
(2) The diffusion bonding interface gap can be quantitively predicted by the correlation curve of the diffusion bonding interface gap and the transducer frequency, and the curve was obtained by the response model for diffusion bonding interface defects.
(3) The diffusion bonding interface gap was verified by the metallographic experiment, and the method proposed was applied to different thicknesses of solid-state welds, including dissimilar materials, providing a very effective method for the prediction of the detectability of diffusion bonding interface defects.

## Figures and Tables

**Table 1:** Device parameters.

Device	Parameters
Transducer	Diameter: 13 mm Frequency: 15 MHz Focus Length: 50 mm
ZEISS Axioscope upright light microscope	(Objective lens) 10 × 100 (Eyepiece lens)
PRC50 ultrasonic board	Frequency range: 0~50 MHz
AL12250 acquisition card	Maximum sampling frequency: 0~250 MHz Storage: 128 M
STAIBLI RX160 robot	Degrees of freedom: 6 Rated load: 20 kg Maximum load: 34 kg Working radius: 1710 mm Repeat positioning accuracy: ±0.05 mm Protection class: IP65

**Table 2:** Acoustic attenuation coefficient.

MHz	dB/mm
5	0.08
10	0.16
15	0.17

**Table 3:** Transducer parameter.

Group Number	Frequency (MHz)	Diameter (mm)	Focus Length (mm)
1	5	13	50
2	10	13	50
3	15	13	50
4	20	13	50

## Data Availability

The data presented in this study are available on request from the corresponding author.
